# Molecular Modeling Insights into Upadacitinib Selectivity upon Binding to JAK Protein Family

**DOI:** 10.3390/ph15010030

**Published:** 2021-12-25

**Authors:** Amir Taldaev, Vladimir R. Rudnev, Kirill S. Nikolsky, Liudmila I. Kulikova, Anna L. Kaysheva

**Affiliations:** 1 Biobanking Group, V.N. Orekhovich Institute of Biomedical Chemistry, 109028 Moscow, Russia; t-amir@bk.ru (A.T.); v.r.rudnev@gmail.com (V.R.R.); glucksistemi@gmail.com (K.S.N.); likulikova@mail.ru (L.I.K.); 2 Department of Chemistry, Sechenov First Moscow State Medical University (Sechenov University), 119991 Moscow, Russia; 3 Institute of Theoretical and Experimental Biophysics, Russian Academy of Sciences, 142290 Pushchino, Russia; 4 Institute of Mathematical Problems of Biology RAS—The Branch of Keldysh Institute of Applied Mathematics of Russian Academy of Sciences, 142290 Pushchino, Russia

**Keywords:** upadacitinib, Janus kinase, JAK inhibitor, rheumatoid arthritis, molecular modeling

## Abstract

Rheumatoid arthritis (RA) is a chronic disease characterized by bone joint damage and incapacitation. The mechanism underlying RA pathogenesis is autoimmunity in the connective tissue. Cytokines play an important role in the human immune system for signal transduction and in the development of inflammatory responses. Janus kinases (JAK) participate in the JAK/STAT pathway, which mediates cytokine effects, in particular interleukin 6 and IFNγ. The discovery of small molecule inhibitors of the JAK protein family has led to a revolution in RA therapy. The novel JAK inhibitor upadacitinib (Rinvoq^TM^) has a higher selectivity for JAK1 compared to JAK2 and JAK3 in vivo. Currently, details on the molecular recognition of JAK1 by upadacitinib are not available. We found that characteristics of hydrogen bond formation with the glycine loop and hinge in JAKs define the selectivity. Our molecular modeling study could provide insight into the drug action mechanism and pharmacophore model differences in JAK isoforms.

## 1. Introduction

Janus kinases (JAKs) are tyrosine kinases associated with cytoplasmic regions of type I and II cytokine receptors. Receptor multimerization occurs when a ligand binds to the receptor [1]. JAKs are involved in the JAK/STAT signal transducer and activator of transcription (STAT) signaling pathway, activation of the immune system, cytokine receptors, and polarization of T-helper cells [1]. This signaling pathway is regulated by a variety of factors, including suppressor of cytokine signaling, protein STAT inhibitors, and protein tyrosine phosphatase, which determine the initiation, duration, and termination of signaling cascades. Dysregulation of the JAK/STAT pathway in T-helper cells can lead to immune disorders [1].

The human JAK family includes four isoforms: JAK1 (P23458), JAK2 (O60674), JAK3 (P52333), and Tyrosine kinase 2 (TYK2, P29597), with molecular weights ranging from 120 kDa to 140 kDa [2]. Each JAK isoform has several domains. The N-terminal domain of FERM (F for 4.1 protein, E for ezrin, R for radixin, and M for moesin) consists of the F1, F2, and F3 subdomains, which structurally resemble the domains that bind ubiquitin and CoA and pleckstrin-phosphotyrosine, respectively [3]. FERM is responsible for protein–protein interactions, such as interactions with membrane-bound proteins [4]. The second SH2 (Src homology 2) domain contains 100 amino acid residues. The SH2 domain activates and dimerizes STAT proteins [5]. The central pseudokinase domain is homologous to the protein tyrosine kinase domain; however, it lacks a catalytic function and plays a regulatory role [6]. Finally, the conserved protein tyrosine kinase domain is located at the C-terminus and contains 250–300 amino acid residues and an ATP-binding site in the immediate vicinity of the catalytic region. The domain is responsible for the phosphorylation of tyrosine residues in target proteins [7].

For all the JAK isoforms, a fairly high level of amino acid sequence identity was observed, which amounted to 22.7% (287 amino acid residues, Clustal Omega [8] multiple alignment algorithm). The highest similarity was observed in the isoforms JAK2 and JAK3, as well as JAK1 and TYK2.

The activation of the JAK/STAT signaling pathway by proinflammatory cytokines is crucial in the pathogenesis and progression of rheumatoid arthritis (RA) [1,9]. Normally, the JAK/STAT signaling pathway is inhibited by a negative linkage mechanism, including suppressors of cytokine signaling and the protein STAT inhibitor. However, in RA, neither of these regulators is involved. Continuous activation of JAK/STAT signaling in synovial joints in RA leads to an increase in the expression level of the matrix metalloproteinase gene, frequency of apoptotic chondrocytes, and resistance to apoptosis in the inflamed synovial tissue [10].

Symptoms of RA, including pain, are mediated by inflammatory and non-inflammatory mechanisms and negatively affect quality of life [10]. Recently published data from a Phase III clinical trial (RA-BEAM; NCT01710358) showed that RA patients treated with JAK1 and JAK2 inhibitors achieved significantly higher pain relief than patients treated with a tumor necrosis factor blocker [10]. Stronger pain relief upon inhibition of JAK1 and JAK2 may reflect the effect on several cytokines involved in the regulation of pain in RA, as opposed to the effect on a single cytokine in the case of TNF blocking [10].

Synthetic JAK inhibitors represent a relatively new class of low-molecular-weight oral drugs, and offer an alternative to RA patients who are unresponsive to conventional or biological therapies. Upadacitinib is a JAK inhibitor with a high selectivity for JAK1 [10]. The drug was recently approved by the US Food and Drug Administration and the European Medicines Agency for use in patients with moderate to severe RA [1].

**Figure 1:**
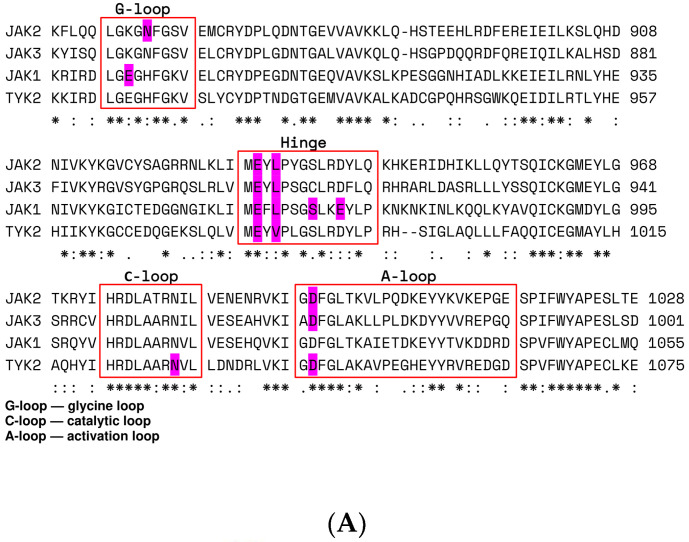

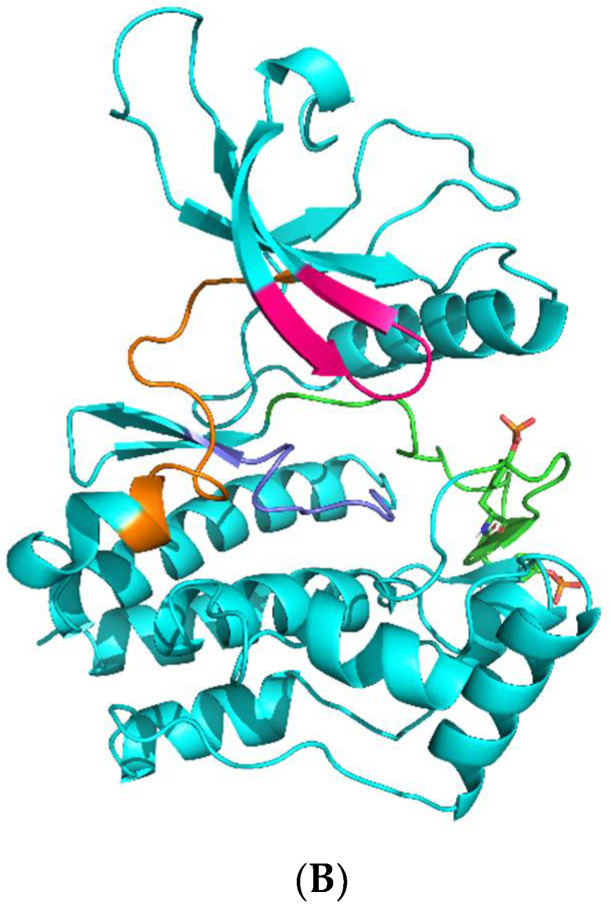
(**A**): JAK kinase domain amino acid sequence alignment was performed in Clustal Omega [8] on the UniProt webserver (*, :, ., and space symbols means 100%, 75%, 50%, 0% match between sequences, respectively). Significant subdomains are highlighted in red. Key amino acid residues that participate in upadacitinib binding are highlighted by magenta. (**B**): JAK kinase domain organization. Glycine loop, hinge, and catalytic and activation loops are colored in magenta, orange, dark blue, and green. Phosphorylated Y residues of the activation loop are shown as sticks.

JAK inhibitors act as competitive inhibitors of ATP and prevent it from binding to the JAK tyrosine kinase domain. Upadacitinib was developed to selectively inhibit ATP binding to JAK1 based on structural differences in the ATP-binding site between JAK1 and JAK2 [1]. The active site of JAKs is formed by several subdomains (Figure 1A,B). These are the β-hairpin of the glycine loop, disordered regions of the hinge, and catalytic and activation loops. Upadacitinib selectively targets JAK1-dependent disease factors, such as IL-6 and IFNγ [1].

**Figure 2:**
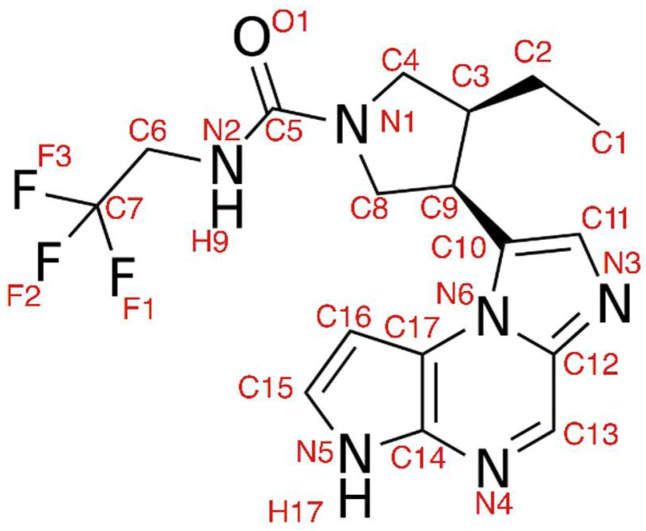
Structural formula of upadacitinib. Internal atom names are shown as red alphanumeric characters.

Upadacitinib contains several condensed heterocycles (imidazole) and is a fluorine derivative of urea (Figure 2). The pharmacophore properties of the molecule are due to its physicochemical characteristics. The molecule can act as a donor of two hydrogen bonds (NH group, hydrogen bond donor) and an acceptor of six hydrogen bonds (hydrogen bond acceptor) according to the number of nitrogen atoms with a lone electron pair. Currently, experimental and computational insights into upadacitinib binding with JAK isoforms are unavailable. The aim of our study was the establishment of structural and dynamic characteristics upon upadacitinib binding to JAK enzyme isoforms that could explain this drug selectivity via molecular modeling methods.

## 2. Results

### 2.1. Molecular Docking

During re-docking, an approximate coincidence of the conformations of co-crystallized and predicted conformations by the criterion of the RMSD (root-mean-square deviation) value was observed during the alignment of the protein-ligand complexes. This allowed us to validate the applicability of the chosen docking path for upadacitinib docking to the active JAK sites (Table 1). The following results were obtained: in the active site, upadacitinib formed three hydrogen bonds with JAK1 (with E883, E957, L959), with JAK2 and JAK3, two hydrogen bonds each (with E930/903 and L932/905, respectively), and only one with TYK2 (V981). An additional hydrogen bond of upadacitinib occurred on the JAK1 glycine loop. A positive correlation was found between the experimental IC50 values and the Vina score (see Table 1). In addition, all native ligands of protein experimental structures showed higher affinity energies than those of upadacitinib. We found that for all compounds, the interaction with amino acid residues of the hinge region, in particular with E and L, was critical. This was a common protein pharmacophore for the binding of JAK inhibitors and experimental crystal structures.

### 2.2. Molecular Dynamics

**Figure 3:**
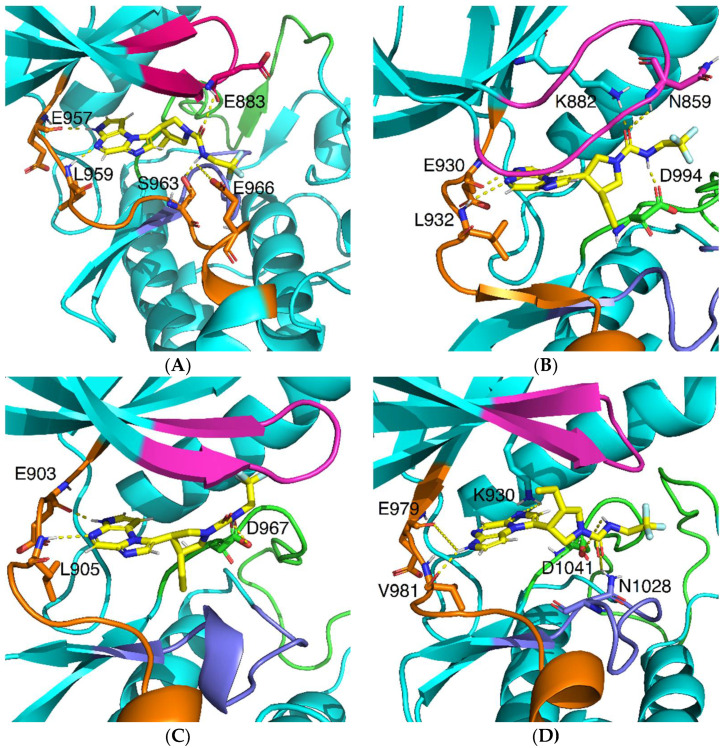
The averaged conformation of upadacitinib from the most populated cluster in the active site of JAKs: (**A**)—in JAK1, (**B**)—JAK2, (**C**)—JAK3, (**D**)—TYK2. Upadacitinib is colored in pseudocolors with yellow carbon atoms. H-bonds are shown as yellow dotted lines. Interactions with upadacitinib amino acid residues are shown as sticks. Specific domains are coded by: glycine loop (magenta), hinge (orange), catalytic loop (dark blue), and activation loop (green).

To confirm the results of molecular docking, we searched for common and unique characteristics in the binding of upadacitinib to JAK isoforms by performing molecular dynamics (MD) simulations of protein-ligand complexes. MD trajectories were efficiently clustered and correlated with the calculated RMSD values for upadacitinib (in all runs, the MD RMSD did not change by more than 0.13 nm) to capture the most preferred modes of drug binding to targets. Figure 3 shows the average of conformations of the protein-ligand complexes generated in the MD trajectory clustering procedure.

#### 2.2.1. Number of Intermolecular Hydrogen Bonds

**Figure 4:**
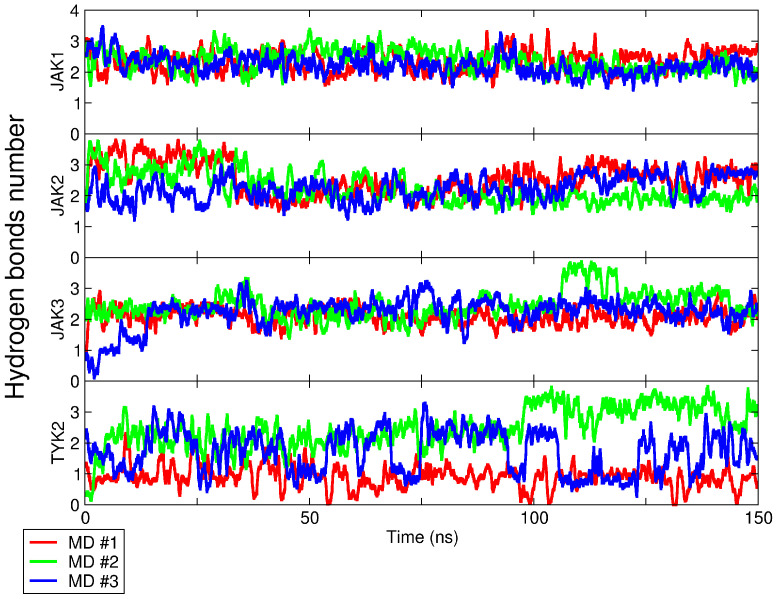
Intermolecular H-bonds number between JAK and upadacitinib during simulation time. Three independent MD runs with each JAK (see Table 4) are shown as colored lines.

The number of intermolecular hydrogen bonds (H-bonds) served as a criterion for the stability of the intermolecular complex during the simulation (Figure 4). It did not change significantly in the JAK isoforms, except in the upadacitinib/TYK2 complex, which may indicate a suboptimal conformation obtained in molecular docking.

For an accurate assessment of intermolecular H-bonds, we calculated them in the most populated cluster. The number of intermolecular H-bonds by upadacitinib in the most populated cluster of MD trajectories was 2.3 ± 0.8, 2.5 ± 1.0, 2.3 ± 0.8, 1.7 ± 1.1 with JAK1, JAK2, JAK3, and TYK2, respectively. The number of H-bonds (JAK2 > JAK1 ≈ JAK3 > TYK2) compared with IC50 values (JAK1 < JAK2 < JAK3 < TYK2; Table 1) did not reveal a direct correlation. This indicates the presence of some characteristics specific to JAK1.

#### 2.2.2. Distribution of Hydrogen Bonds

**Figure 5:**
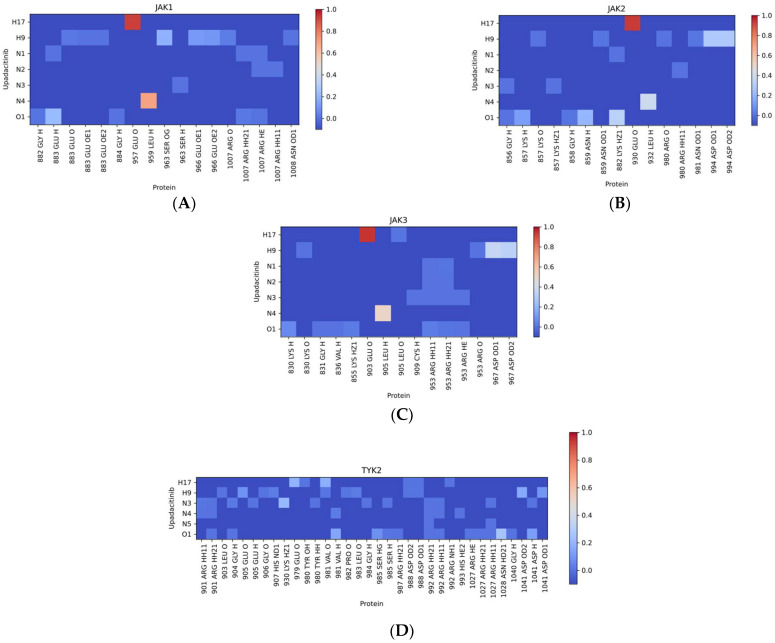
Intermolecular H-bond occurrence heatmaps between interacting upadacitinib and protein atoms. Color bar shows the occurrence in the most populated cluster. (**A**)—upadacitinib/JAK1, (**B**)—upadacitinib/JAK2, (**C**)—upadacitinib/JAK3, (**D**)—upadacitinib/TYK2.

The intermolecular H-bonds were analyzed in detail. We generated a heatmap for H-bonds arising between upadacitinib and JAK isoforms, and calculated the occurrence of non-covalent interactions. All kinases interacted with the hinge region (Figure 5A–D). However, four H-bonds with hinge JAK1 (E957 backbone oxygen, L959 backbone amide proton, S963 sidechain oxygen, and E966 sidechain oxygens) were found in upadacitinib (Figure 5A), versus two with JAK2 (E930 backbone oxygen, L932 backbone amide proton) (Figure 5B), JAK3 (E903 backbone oxygen, L905 backbone amide proton) (Figure 5C), and TYK2 (E979 and V981 oxygen atoms) (Figure 5D). For TYK2, the binding of the hinge region significantly differed, which was confirmed experimentally [11], wherein, in another small molecule, binding occurred with V981. Moreover, upadacitinib interacted with the glycine loop in JAK1 (E883 backbone oxygen) and JAK2 (N859 backbone amide proton). No H-bond formation was observed in the glycine loops of other JAKs. Thus, the general pattern of binding to E and L amino acid residues of the hinge JAKs was confirmed; however, only the MD experiment revealed the unique property of the protein pharmacophore JAK1.

#### 2.2.3. Changes in the JAK Structure upon Binding of Upadacitinib

**Figure 6:**
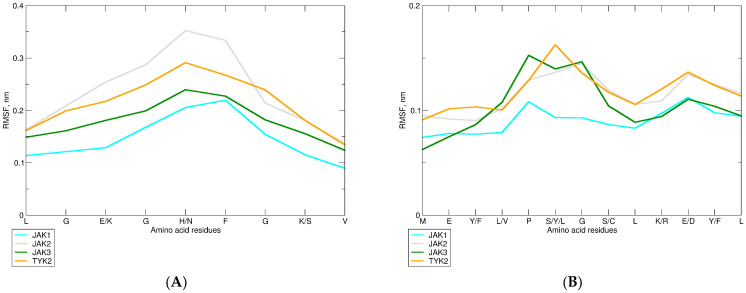
RMSF plots of Cα atoms of JAKs’ glycine loop (**A**) and hinge (**B**) subdomains in the most populated cluster. Colored lines show the protein atoms of each JAK.

We selected changes in the RMSF (root-mean-square fluctuations) Cα atoms of amino acid residues as a measure of the mobility of JAK subdomains upon binding of upadacitinib. Interaction with upadacitinib reduced glycine loop mobility in JAK1 and JAK2 compared to other kinases (Figure 6A). Moreover, additional H-bonds reduced the hinge mobility of JAK1 (Figure 6B). In contrast, only one intermittent H-bond of TYK2 bound with upadacitinib correlated with the highest hinge mobility of this kinase.

#### 2.2.4. Free Binding Energy Estimation via MM-PBSA Method

Free binding energies (ΔG_bind_) of upadacitinib with JAKs were distributed as follows: JAK1 < JAK3 < JAK2 < TYK2 (Table 2). The free binding energy does not directly correlate with the experimental IC50 values because of the estimation of the entropy contribution in the experimental and calculation methods. However, the binding energy of the drug to JAK1 was the most favorable. Additional information can be obtained from contributions of the binding energy. Thus, the energy of van der Waals interactions was lower for JAK1 compared to other JAKs. The catalytic and activation loops formed a hydrophobic pocket. However, the van der Waals interaction was only present in upadacitinib/JAK1 binding, where L1010 was important. The highest energy of electrostatic interaction was observed for TYK2 with upadacitinib. This correlated with the low number of intermolecular H-bonds (Figure 5D).

## 3. Discussion

The JAK family of human enzymes includes JAK1, JAK2, JAK3, and TYK2 isoforms. JAK enzymes are important molecules for cytokine and growth factor signaling [1]. Isoforms of the JAK family function in combination; however, under certain conditions, independent activity of a JAK isoform may prevail over another. A striking example of the latter is signaling via the proinflammatory mediator IL-6 in RA. IL-6 leads to the activation of all JAK isoforms; however, a dominant role is observed in JAK1 [2]. JAK3, together with JAK1, is an important component in signaling for common gamma cytokine receptors IL-2, IL-4, IL-7, IL-9, IL-15, and IL-21 [3]. These cytokines are involved in many physiological processes, including T cell survival, Th2 responses, and natural killer (NK) cell survival. JAK2 is involved in the regulation of hematopoietic cell differentiation through signaling via IL-3, IL-5, granulocyte-macrophage colony-stimulating factor, erythropoietin, thrombopoietin, growth hormone, and leptin. TYK2 is involved in the differentiation of Th1 and Th17 cells through IL-12 and IL-23 [4].

JAK enzymes play an important role in the pathogenesis of RA [5]. Although the molecular mechanisms of RA pathogenesis remain unelucidated, the disease is accompanied by dysregulation of immune responses and production of autoantibodies and cytokines. Monocytes, T cells, and B cells play an important role in the pathogenesis of RA [6]. Dysregulation of the JAK/STAT signaling pathway in circulating immune cells mediates a chronic inflammatory response [5,7].

To date, tofacitinib, baricitinib, pecitinib, upadacitinib, and filgotinib, which are JAK enzyme inhibitors, have been developed and are actively used in pro-rheumatic therapy [4]. JAK inhibitors are targeted low-molecular-weight synthetic disease-modifying antirheumatic drugs. JAK inhibitors suppress the action of intracellular JAK kinases [4]. Padacintinib is a selective inhibitor of JAK1. The selectivity of the interaction of upadacitinib predominantly with the JAK1 isoform was confirmed by experimental studies. The results of the enzymatic studies confirmed the high selectivity of upadacitinib for JAK1. IC50 values were obtained for the JAK isoforms, indicating a decrease in the selectivity for the binding of the pharmacophore/enzyme in decreasing order of JAK1 > JAK2 > JAK3 > TYK2 [9].

In the present study, assessing the molecular dynamics, we characterized features of the geometry of the active binding sites of the JAK enzymes and mapped amino acid residues involved in the formation of the complex with upadacitinib. Further, we explained the binding selectivity of upadacitinib to JAK1 using the following observations.

We developed structural models for the four JAK isoforms using experimental data from the PDB database (Table 3). The amino acid sequences of proteins of the JAK family were highly conserved; the homology of amino acid residues in the binding site of the pharmacophore for JAK1 and JAK2 was 85% (Figure 1). The formation of H-bonds with E and L is a common interaction with the hinge region in all previously studied isoforms. Previously, the work of Michael Friedman’s scientific group revealed that the glycine loop in the binding site of the JAK1 pharmacophore forms a closed conformation, in contrast to JAK2. This could be attributed to differences in the amino acid sequences [10]. Parmentier performed a structural analysis of the binding sites of JAK1 and JAK2, which showed that the trifluoroethyl group occupies the space under the glycine loop [1].

We obtained structural models of binding sites for the four isoforms of JAK and identified structural features that were not previously characterized. Our results indicated that in the active sites of JAK1 and TYK2, the 2,2,2-trifluoroethyl group of upadacitinib was not located under the glycine loop (Figure 5A,D). In contrast, JAK2 and JAK3 showed the possibility of the 2,2,2-trifluoroethyl group being located under the glycine loop. This behavior was similar to that of the analog compounds used in this study [12]. There were obstructions created by the imidazole cycle of histidine in the glycine loop during visual inspection of the MD trajectories. In place of histidine in the glycine loop, there was an E, which did not create such steric hindrances, and the 2,2,2-trifluoroethyl group could be located there. A more accurate assessment of this impact will be required in the future. In addition, molecular docking and MD experiments revealed a different binding mode to TYK2. Upadacitinib was “flipped” 180 degrees compared to the pose in the other kinases studied.

This study evaluated the binding energies of upadacitinib with JAKs. We assume that some inaccuracy exists in the estimation of free binding energies; however, calculations of some contributions to binding energy have been used to characterize the protein pharmacophore. For the most accurate estimate of ΔG_bind_, methods with the calculation of the potential of the potential of mean force, or perturbations of free energies, should be used.

## 4. Materials and Methods

### 4.1. Initial Molecular Structure Preparation

The geometrically optimized structure of upadacitinib with unit atoms was obtained from the Automated Topology Builder 3.0 server (Molid: 515309) (accessed on 2 October 2021) [13,14]. Structures of the kinase domain were obtained from the RCSB Protein Data Bank (accessed on 2 October 2021) (Table 3). Heteromolecules (including water) were manually removed using PyMol software (Schrödinger, LLC). Missing residues JAK1 and TYK2 were reconstructed in the SWISS-MODEL server from the primary amino acid sequence of the original structures [15].

### 4.2. Molecular Docking

The protein structures of JAKs, co-crystallized native ligands, and the structure of upadacitinib were prepared for molecular docking in AutoDockTools 1.5.6 by adding partial charges according to the Gasteiger method for all molecules [19] and torsions for small molecules only. Grid maps of the receptor for one of the ligands were generated as 30 × 30 × 30 Å dimensions of a box placed on the active sites of JAKs through *x*, *y*, and *z* coordinates, respectively. Docking was performed in AutoDock Vina 1.1.2 in 10 replicates [20]. The Vina scores and ligand conformations were ranked.

### 4.3. Molecular Dynamics

The best binding modes of upadacitinib in the active sites of JAKs from molecular docking were selected for assessing the molecular dynamics (MD) of protein-ligand complexes. MD simulations were performed using the GROMACS 2020.4 software [21]. The modified GROMOS96 54A7 force field [22] was used with non-standard amino acid residue parameter sets [23]. Upadacitinib topology was obtained from Automated Topology Builder 3.0 (Molid: 515309) [13,14]. DFT/B3LYP/6-31G* quantum mechanics optimization, and Merz–Singh–Kollman partial charges were applied. MD simulations were performed in an explicit solvent under periodic boundary conditions. Each initial structure was centered in a cubic box of sufficient size so that the minimum distance to period images was 1.0 nm. A simple point-charge water model was employed for the simulations. The system was neutralized with Na^+^ ions. The compositions of the built systems are presented in Table 4. Proteins with upadacitinib and non-protein atoms were coupled to their temperature baths set at 311 K using the V-rescale algorithm [24]. The pressure was maintained isotropically at 1 Bar using a Berendsen barostat [25]. A time step of 2 fs was used. Each system underwent energy minimization using the steepest descent algorithm (1000 steps), followed by gradual heating from 5 K to 311 K during a 200 ps MD run with fixed heavy atoms of structural elements. The initial atom velocities were obtained from a Maxwellian distribution at 311 K, and the bond lengths were constrained using LINCS [26]. A 1.4 nm cut-off was used for Lennard-Jones interactions, and dispersion corrections for energy and pressure were applied. Electrostatics were calculated using the particle-mesh Ewald method with a 0.12 nm grid-spacing and a 1.4 nm real-space cut-off. Each trajectory was run for 150 ns in three replicates, with a total simulation time of 1.8 μs.

### 4.4. Molecular Dynamics Trajectories Analysis

The computed MD trajectories were analyzed using the built-in GROMACS software and custom utilities. The trajectories in each run were merged. Preferred conformations of upadacitinib in JAK active sites were determined using the cluster module of the GROMACS package (GROMOS clustering method with cut-off values presented in Table 4). The largest clusters were extracted from the MD trajectories for the subsequent analysis. The dynamic behavior and stability of the studied systems were assessed by root-mean-square deviation (RMSD) and root-mean-square fluctuation (RMSF) calculations. Hydrogen bonds (H-bonds) were analyzed in the GROMACS *hbond* module and in the originally developed software.

### 4.5. Molecular Mechanics Poisson–Boltzmann Surface Area (MM-PBSA) Free Binding Energy Estimation

Two hundred random frames from the most populated cluster were extracted into a new trajectory for each JAK/upadacitinib complex from MD simulations. The molecular mechanics Poisson–Boltzmann surface area (MM-PBSA) method was used for the free binding energy (ΔG_bind_) estimation in the *g_mmpbsa* tool [27].

## 5. Conclusions

In the present study, structural models of the organization of binding sites for the JAK family enzymes and for the pharmacophore model were developed in order to substantiate the selectivity of upadacitinib for JAK1 using experimental structures of JAK proteins. We believe that the highest affinity of upadacitinib was due to the formation of four hydrogen bonds with JAK1 hinge region amino acid residues, against two bonds with other JAK isoforms. We attribute the stabilization of the molecule in the hinge region to structural features of the JAK1 binding site, such as S963 and E966, which are distinctive residues of this tyrosine kinase isoform. The affinity was also positively influenced by the hydrogen bond with the amino acid residues of the glycine loops in JAK1 (E883) and JAK2 (N859) with upadacitinib. Our study can help with the development of selective and efficient next-generation JAK inhibitors, hence, improving the treatment of various cytokine-mediated diseases.

## Figures and Tables

**Table 1:** Results of molecular docking.

Kinase	Co-Crystallized Native Ligand Vina Score, kcal/mol ^1^	RMSD between Re-Docked and Co-Crystallized Ligand, nm	IC50 of Upadacitinib, nM ^2^	Upadacitinib Vina Score, kcal/mol ^1^
JAK1	−9.4	0.007	43	−8.5
JAK2	−9.1	0.006	120	−8.1
JAK3	−9.6	0.009	2300	−7.9
TYK2	−10.9	0.000	4700	−6.0

^1^ Lower value is better. ^2^ Literature data [1].

**Table 2:** Binding energies of upadacitinib with JAKs and contributions to them.

Kinase	van der Waals Energy ^1^, kcal/mol	Electrostatic Energy ^1^, kcal/mol	Polar Solvation Energy ^1^, kcal/mol	SASA Energy ^1,2^, kcal/mol	Binding Energy ^1^, kcal/mol
JAK1	−45.0 ± 3.5	−12.6 ± 3.5	38.8 ± 6.7	−4.4 ± 0.2	−23.2 ± 4.2
JAK2	−39.3 ± 0.3	−21.0 ± 0.4	48.6 ± 0.8	−4.3 ± 0.0	−16.0 ± 0.4
JAK3	−42.4 ± 0.3	−15.7 ± 0.2	41.5 ± 2.8	−4.4 ± 0.0	−20.9 ± 0.3
TYK2	−35.8 ± 0.3	−10.9 ± 0.3	38.2 ± 0.6	−4.1 ± 0.0	−12.6 ± 0.3

^1^ Lower value is better. ^2^ Solvent accessible surface area.

**Table 3:** Information about investigated kinase domains.

Protein Molecule	UniProt ID	PDB ID	Native Ligand	Amino Acids	PTM (Phosphotyrosine)	Reference
JAK1	P23458	6N7A	*N*-[3-(5-chloro-2-methoxyphenyl)-1-methyl-1*H*-pyrazol-4-yl]-2-methyl-2*H*-pyrazolo [4,3-c]pyridine-7-carboxamide	865–1154	1034, 1035	[16]
JAK2	O60674	4YTH	*N*~2~-[2-(5-chloro-1*H*-pyrrolo[2,3-b]pyridin-3-yl)-5-fluoropyrimidin-4-yl]-2-methyl-*N*-(2,2,2-trifluoroethyl)-*D*-alaninamide	842–1130	1007, 1008	[17]
JAK3	P52333	5LWN	(2~{*S*})-2-cyano-~{*N*},~{*N*}-dimethyl-3-[5-[3-[(1~{*S*},2~{*R*})-2-methylcyclohexyl]-3,5,8,10-tetrazatricyclo[7.3.0.0^{2,6}]dodeca-1,4,6,8,11-pentaen-4-yl]furan-2-yl]propanamide	814–1103	-	[18]
TYK2	P29597	3LXP	4-*tert*-butyl-15-fluoro-3,5,10-triazatetracyclo[11.4.0.02,6.07,12]heptadeca-1(13),2(6),4,7(12),8,14,16-heptaen-11-one	888–1178	1054	[11]

**Table 4:** System composition and analysis details.

Simulated System Composition	MD Duration, ns	Clustering Cut-Off Values, nm
JAK1		
Protein(1)/Upadacitinib(1)/Water(11173)/Na^+^(4)	3 × 150	0.10
JAK2		
Protein(1)/Upadacitinib(1)/Water(10721)/Na^+^(4)	3 × 150	0.12
JAK3		
Protein(1)/Upadacitinib(1)/Water(10919)	3 × 150	0.12
TYK2		
Protein(1)/Upadacitinib(1)/Water(11456)/Na^+^(7)	3 × 150	0.10

## Data Availability

Data is contained within the article.
